# Non-Palladium-Catalyzed Oxidative Coupling Reactions Using Hypervalent Iodine Reagents

**DOI:** 10.3389/fchem.2022.909250

**Published:** 2022-07-01

**Authors:** Samata E. Shetgaonkar, Aleena Raju, Hideyasu China, Naoko Takenaga, Toshifumi Dohi, Fateh V. Singh

**Affiliations:** ^1^ Chemistry Division, School of Advanced Science, VIT University, Chennai, India; ^2^ Department of Medical Bioscience, Nagahama Institute of Bio-Science and Technology, Nagahama, Japan; ^3^ Faculty of Pharmacy, Meijo University, Nagoya, Japan; ^4^ College of Pharmaceutical Sciences, Ritsumeikan University, Kusatsu, Japan

**Keywords:** hypervalent iodine reagents, oxidative coupling, gold, copper, oxidant, catalyst

## Abstract

Transition metal-catalyzed direct oxidative coupling reactions via C–H bond activation have emerged as a straightforward strategy for the construction of complex molecules in organic synthesis. The direct transformation of C–H bonds into carbon–carbon and carbon–heteroatom bonds renders the requirement of prefunctionalization of starting materials and, therefore, represents a more efficient alternative to the traditional cross-coupling reactions. The key to the unprecedented progress made in this area has been the identification of an appropriate oxidant that facilitates oxidation and provides heteroatom ligands at the metal center. In this context, hypervalent iodine compounds have evolved as mainstream reagents particularly because of their excellent oxidizing nature, high electrophilicity, and versatile reactivity. They are environmentally benign reagents, stable, non-toxic, and relatively cheaper than inorganic oxidants. For many years, palladium catalysis has dominated these oxidative coupling reactions, but eventually, other transition metal catalysts such as gold, copper, platinum, iron, etc. were found to be promising alternate catalysts for facilitating such reactions. This review article critically summarizes the recent developments in non-palladium-catalyzed oxidative coupling reactions mediated by hypervalent iodine (III) reagents with significant emphasis on understanding the mechanistic aspects in detail.

## Graphical Abstract

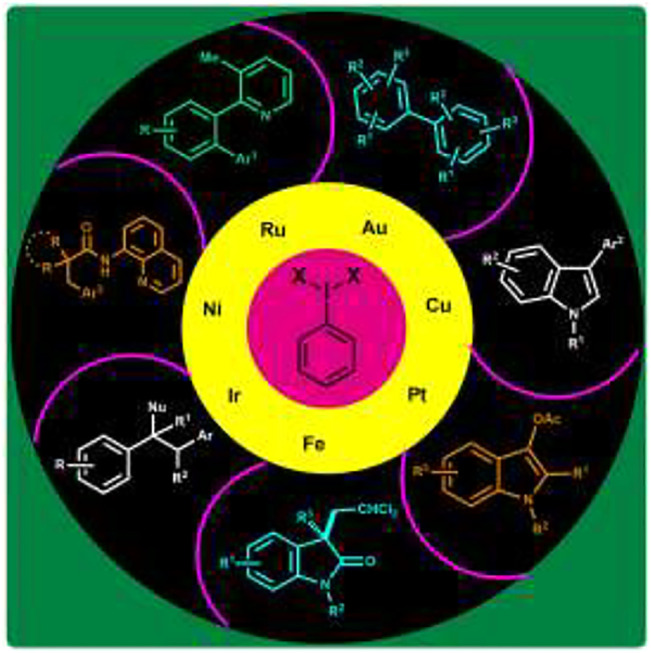

## Introduction

Transition metal-catalyzed cross-coupling reactions have emerged as a powerful synthetic method for the construction of valuable organic molecules (Jana et al., 2011; Campeau, and Hazari, 2019). These represent a versatile strategy to connect nucleophilic organometallic compounds with electrophilic organic halides in the presence of palladium or other transition metal catalysts. A wide range of nucleophiles such as organo-boron, organo-zinc, and Grignard reagents have been used as coupling partners in these reactions (Jana et al., 2011; Campeau, and Hazari, 2019). However, a few drawbacks associated with traditional cross-coupling reactions include pre-activation of substrates and stoichiometric requirement of organometallic reagents that generate metallic salts as by-products leading to severe environmental concerns. From a green chemistry point of view, continuous efforts were directed toward the development of direct oxidative coupling reactions that could eliminate the requirement of prefunctionalization of substrates and also minimize waste material generation. In this aspect, new oxidative coupling reactions via transition metal-catalyzed direct C–H bond functionalization have evolved as a more efficient alternative to traditional cross-coupling reactions (Kuhl et al., 2012; Qiu and Wu, 2015; Henry et al., 2017). This method has enabled the effective formation of new carbon–carbon and carbon–heteroatom bonds by coupling non-prefunctionalized substrates via the C–H bond activation method. The key to the unprecedented progress made in this area has been the identification of appropriate catalysts, oxidants, additives, and ligands. Hypervalent iodine reagents have gained significant attention in this field owing to their excellent oxidizing and electrophilic properties.

**FIGURE 1:**
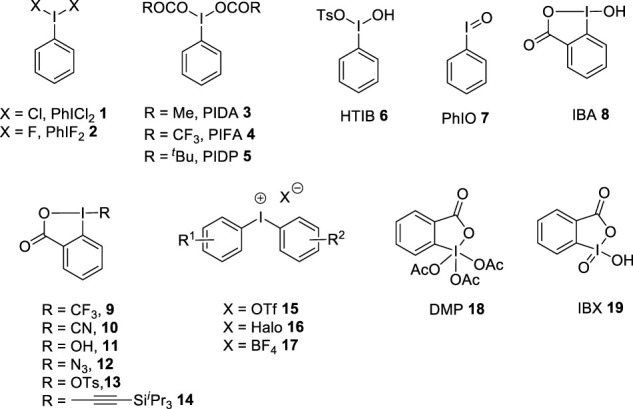
Hypervalent iodine (III)/(V) reagents **1**–**19**.

Hypervalent iodine reagents are of utmost importance in modern organic synthesis as they are environmentally benign alternatives for toxic heavy metal oxidants (Dohi and Kita, 2009; Silva and Olofsson, 2011; Yusubov and Zhdankin, 2012; Kita and Dohi, 2015; Yoshimura and Zhdankin, 2016). They are highly stable, less toxic, readily available, easy to handle, and possess the ease of recovery from the reaction system. Various iodine(III) and iodine(V) reagents are existing in the literature and the structures of few representative examples are given in Figure 1. The first hypervalent iodine reagent, namely, (dichloroiodo)benzene **1** was prepared by Willgerodt (1886). After this, several iodine (III) compounds such as (difluoroiodo)benzene **2**, phenyliodine (III) diacetate **3** (PIDA), phenyliodine (III) bis(trifluoroacetate) **4** (PIFA), phenyliodine (III) dipivaloate **5** (PIDP), [hydroxy (tosyloxy)iodo]-benzene HTIB **6** (Koser′s reagent), iodosylbenzene **7** (PhIO), 2-iodosobenzoic acid **8** (IBA), benziodoxol (on)e reagents **9**–**14**, and diaryliodonium salts **15**–**17** were identified as powerful and effective oxidants and atom transfer reagents in different organic transformations (Tohma and Kita, 2004; Joshi and De, 2021). In addition to this, cyclic iodine(V) reagents, namely, Dess–Martin periodinane (DMP) **18** and 2-iodoxybenzoic acid (IBX) **19** are strong oxidizing agents (Uyanik and Ishihara, 2009). Several studies (Singh and Wirth, 2014a; Elsherbini and Wirth, 2018; Grelier et al., 2018; Singh et al., 2018; Hyatt et al., 2019; Shetgaonkar and Singh 2020; Shetgaonkar et al., 2021; Shetgaonkar et al., 2021; Singh and Wirth, 2021; Kumar et al., 2022) and books chapters (Singh and Wirth, 2014b; Singh and Wirth, 2017; Singh and Wirth, 2018) have been published covering the preparations and synthetic applications of these reagents.

Undoubtedly, hypervalent iodine reagents have led to the rapid growth in transition metal-catalyzed oxidative coupling reactions. In particular, they are used as selective oxidants and as ligand transfer reagents in these reactions. Hypervalent iodine reagents as oxidants facilitate the desired oxidation at the metal center and also provide heteroatom ligands such as acetate or chloride which are subsequently transferred to the substrates via reductive elimination. To date, palladium has been exclusively used as a catalyst in various oxidative coupling reactions involving the direct functionalization of C (sp^2^)–H and C (sp^3^)–H bonds (Deprez and Sanford, 2007; Silva et al., 2017; Shetgaonkar and Singh, 2020). On the other hand, other transition metals do catalyze such oxidative coupling reactions in the presence of hypervalent iodine reagents. To the best of our knowledge, studies on hypervalent iodine-mediated non-palladium-catalyzed oxidative coupling reactions remain rare (Silva et al., 2017). In the present review article, we will cover the recent advancements made in non-palladium-catalyzed oxidative coupling reactions using hypervalent iodine reagents. The article is classified on the basis of metal catalysts used such as gold, copper, platinum, iron, iridium, nickel, and ruthenium.

## Gold

Gold has contributed a significant role in the development of oxidative coupling reactions. This is primarily because of its excellent ability as a carbophilic activator over other transition metals. Gold catalysis follows redox neutral pathways involving Au(I)/Au(III) catalytic cycles. Typically, reactions involving Au(I)/Au(III) catalytic cycles are rare because of the high barrier for the oxidation of Au(I) species to the Au(III) species (redox potential +1.41 V). Thus, the identification of powerful oxidants which can facilitate the oxidation of Au(I) to Au(III) would be crucial. In this context, hypervalent iodine reagents have emerged as the choice of oxidants because of their excellent oxidizing nature and high electrophilicity. This has led to the development of gold-catalyzed homo/cross-coupling, alkynylation, and alkene functionalization reactions using hypervalent iodine reagents as excellent oxidants and electrophilic functional group transfer reagents. In this section, we will highlight some of the significant studies accomplished in this area in great detail.

**FIGURE 2:**
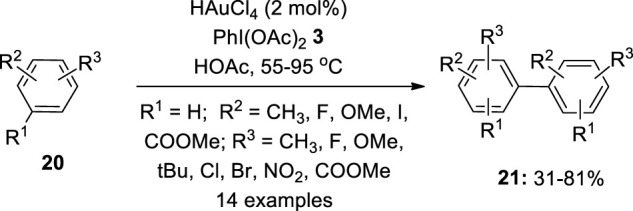
Gold-catalyzed oxidative coupling of arenes **20** for the synthesis of biaryls **21** using PhI(OAc)_2_ **3** as an oxidant.

Gold-catalyzed cross-coupling reaction of arenes is an interesting method for the construction of biaryls. Although this area is less developed, a few examples have been reported using a hypervalent iodine reagent as an oxidant. In 2008, Tse et al. first reported a gold-catalyzed oxidative coupling of arenes **20** to obtain biaryls **21** via an electrophilic aromatic substitution pattern (Kar et al., 2008) (Figure 2). The reaction yielded homo-coupling products **21** in mild conditions using PhI(OAc)_2_ **3** as an oxidant. The process used a variety of gold catalysts, including HAuCl_4_, Au(OAc)_3_, AuCl(PPh_3_), and generated products **21** with 74, 39, and 76% yields respectively. When acetic acid was used as a solvent, the process proceeded smoothly. Non-coordinative solvents, such as 1,2-dichloroethane, can also be used as a reaction medium. Notably, the present reaction does not require an inert environment, a pre-treated substrate, or silver salts to boost reactivity, and it can run under normal conditions. Furthermore, even if the starting material is used at a higher concentration, it can be reused. Arenes **20** containing diverse functional groups, such as esters and methoxides, were also tolerated. For halogenated arenes **20**, homo-coupled products **21** are formed that are not fulfilled by Lewis acid or Pd-catalyzed reactions. It also produced considerable yields of electron-rich heterocycles such as thiophenes. The gold-mediated homo- and hetero-coupling of non-activated arenes was explored later by the same group (Kar et al., 2009).

**FIGURE 3:**
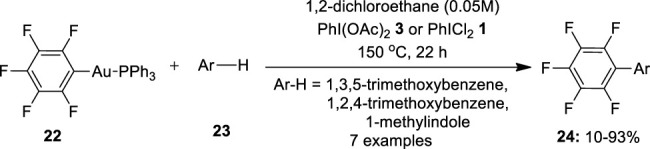
Gold-catalyzed synthesis of biaryls **24** via oxidative coupling of pentafluorophenyl gold(I) complexes **22** with electron-rich arenes **23** using PhI(OAc)_2_ **3** as an oxidant.

Another gold-based cross-coupling reaction of arenes was reported by Hofer and Nevado (2013). In this approach, they performed oxidative coupling of pentafluorophenyl gold(I) complexes **22** with electron-rich arenes **23** such as 1,3,5-trimethoxybenzene, 1,2,4-trimethoxybenzene, or 1-methylindole in the presence of hypervalent iodide oxidants such as PhI(OAc)_2_ **3** and PhICl_2_ **1** (Figure 3). Surprisingly, the complex generated a hetero-coupling product **24** with a 67% yield when treated with 1,3,5-trimethoxybenzene in the presence of PhI(OAc)_2_ **3**. Similarly, the reaction of **22** with 1,2,4-trimethoxybenzene gave 76% of hetero-coupling product **24** whereas with 1-methylindole, it yielded 80% of the product. In the absence of an oxidant, the reaction produced no product. Further replacing the oxidant with PhICl_2_ **1** resulted in albeit lower yields of the hetero-coupled products **24**. The stronger basic character of AcO- was posited as the reason for this alteration in the reaction pattern. Later, Cambeiro et al. (2013)designed a similar oxidative coupling method involving double C–H activation for the synthesis of biaryls by using PhI(OH)OTs **6** as an oxidant.

**FIGURE 4:**
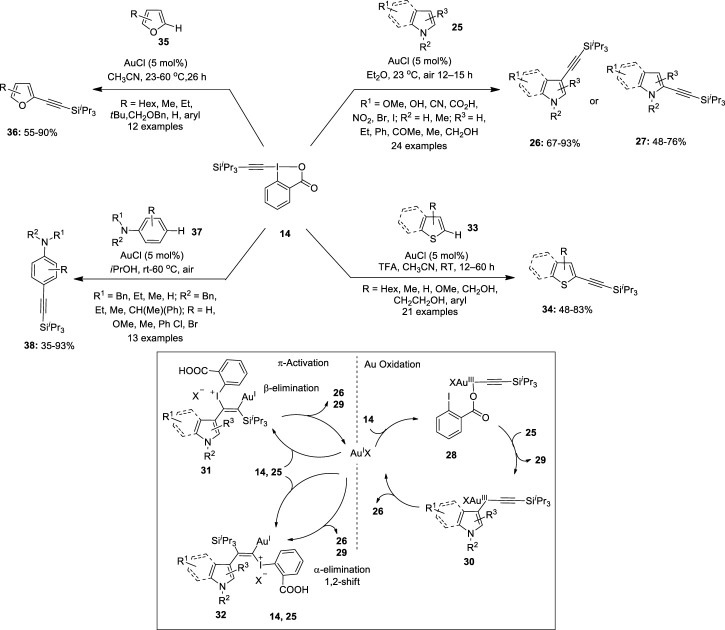
Gold-catalyzed C–H alkynylation of arenes/hetero-arenes using a benziodoxolone-derived hypervalent iodine reagent **14** as an acetylene transfer reagent.

Further hypervalent ethynyl benziodoxolone (EBX) reagents have evolved as a mainstream reagent in various gold-catalyzed electrophilic functional group transfer reactions (especially alkynylations, acetoxylations, and arylations) (Banerjee et al., 2020). The unique electrophilicity and oxidizing nature of these reagents have led to the rapid development in the direct C–H alkynylation reactions of (hetero)aromatic compounds using gold catalysis. In 2009, Waser et al. reported the first gold-catalyzed C–H alkynylation of indoles and pyrroles **25** with 1-[(triisopropylsilyl)ethynyl]-1,2-benziodoxol-3(1*H*)-one (TIPS-EBX) **14** for acetylene transfer (Figure 4) (Brand et al., 2009). The reaction proceeded in high yields using 5 mol% AuCl in Et_2_O at room temperature in air. Several indole derivatives were alkylated to deliver corresponding 3-alkynylation products **26** in good yields. Both electron-donating and electron-withdrawing groups were tolerated in the reaction. Interestingly, higher yields were obtained for substrates with Br and I substituents. Also, 2-substituted indoles **25** gave good yields of 3-alkynylation products **26**. Moreover, the present protocol was successfully used for the alkynylation of 3-methylindole **25** to yield 76% of the 2-alkynylation product **27**. Further alkynylation of pyrroles **25** was achieved for the first time under the same reaction conditions. Mono-, di-, and tri-substituted pyrroles **25** yielded 2-alkynylation products **27** in good yields. The two working hypotheses for the mechanism of the alkynylation reaction are depicted in Figure 4. In one hypothesis, the oxidation of Au(I) with **14** to give a Au(III)–acetylene complex **28**, followed by indole metalation and reductive elimination yields a 3-alkynylation product **26**. The other hypothesis involves Au-mediated addition of indole **25** to the triple bond of **14** to provide a vinyl-gold complex **31** or **32** followed by either β-elimination or α-elimination/1,2-shift sequence to give the desired alkynylation product **26**.

Later, Brand and Waser (2010)demonstrated the alkynylation of thiophenes **33** by using benziodoxolone-derived hypervalent iodine reagent **14** as an acetylene transfer reagent (Figure 4). Notably, the activation of benziodoxole reagent **14** using a gold catalyst (AuCl) and Brønsted acids (TFA) was required for the success of this reaction. 2-Alkyl or 2-aryl-substituted thiophenes **33** were alkynylated in good yields. Moreover, mono-alkynylation of 2,2′-bithiophenes was achieved to yield important building blocks for oligothiophenes. In addition, alkynylation of less reactive benzothiophenes **33** was also investigated; however, no regioselectivity was observed and mixtures of 2- and 3-alkynylated thiophenes **34** were obtained in 65% yield. In continuation, Waser et al. reported the first example highlighting the direct alkynylation of furans **35** by using a Au(I) catalyst and TIPS-EBX **14** (Li, et al., 2013) (Figure 4). Initially, the standard conditions for thiophene alkynylation (TFA and acetonitrile) were used; however, it led to the decomposition of the starting material possibly due to the acid sensitivity of furan moiety. Thus, C2-alkynylation of furans **35** was performed in the absence of TFA and the desired alkynyl furans **36** were isolated in useful yields. Notably, this reaction was found to be highly selective toward the electron-rich C2 position of the furan ring. The alkynylation of 2-alkyl-substituted furans **35** proceeded well at room temperature to give 2-alkynylated products **36** in decent yields. Conversely, the reaction of 2-aryl furans **35** was slower and the corresponding 2-alkynylated furans **36** were isolated under heating conditions. Moreover, 2,5-disubstituted furan **35** yielded a 3-alkynylation product **36** in 45% yield.

Furthermore, Brand and Waser (2012)used TIPS-EBX **14** as an alkynylation reagent for the *para*-selective alkynylation of anilines **37** using a gold catalyst (Brand and Waser, 2012) (Figure 4). ^*i*^PrOH as a solvent system provided the best result for the alkynylation reaction. A variety of anilines **37** were alkynylated regioselectively to the corresponding *para*-alkynyl anilines **38** in moderate to excellent yields. Interestingly, anilines **37** protected with benzyl, methyl, ethyl, and butyl groups as nitrogen substituents were tolerated. Moreover, the alkynylation of mono-protected anilines **37** was successful, indicating a tolerance toward free NH bonds on the aniline. Importantly, dibenzylated anilines **37** with *meta*-substitutions yielded 1,3,4-substituted anilines **38** in useful yields. In addition, the reaction attempted with a *para*-substituted aniline yielded an *ortho*-alkynylated product in 18%.

**FIGURE 5:**
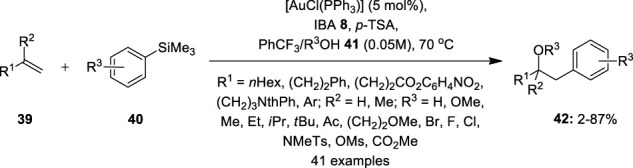
Gold-catalyzed oxyarylation of mono- and gem-di-substituted olefins **39** using IBA **8** as an oxidant.

A gold-hypervalent iodine combination was further explored for the hetero-arylation of olefins **39** by Ball et al. (2012). They designed a three-component oxyarylation reaction by using a gold catalyst and 1-hydroxy-1,2-benziodoxol-3(1*H*)-one (IBA) **8** as an efficient oxidant (Figure 5). The reactions required the presence of *p*-TSA as an additive. Notably, benzotrifluoride (PhCF_3_) as solvent media substantially reduced the formation of homo-coupling products. A range of mono-substituted olefins **39** smoothly reacted with diverse arylsilanes **40** in the presence of alcohols **41** as *O*-nucleophiles to deliver corresponding products **42** in variable yields. *O*-nucleophiles such as methanol, ethanol, isopropanol, and water could be used, giving desired products in moderate yields. Arylsilanes **40** with electron-rich and electron-deficient substituents were well tolerated. However, electron-rich arylsilanes, carboxylic acids, and tertiary alcohols were unsuited to this reaction. Further oxyarylation of styrenes and gem-di-substituted olefins proceeded smoothly under similar conditions, although the reaction scope was limited to MeOH as an *O*-nucleophile.

**FIGURE 6:**
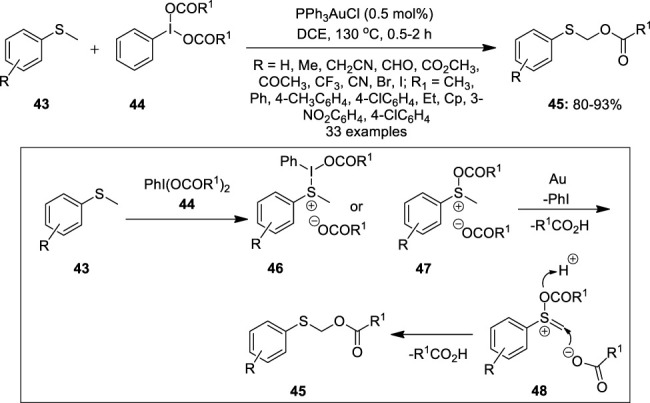
Au(II)-catalyzed α-C (sp^3^)–H acyloxylation of methyl sulfides **43** using bis(acyloxy)iodobenzene **44** as an oxidant.

Further significant work on the oxidative acyloxylation of an unactivated C (sp^3^)–H bond was achieved by Yang et al by gold catalysis (Guo et al., 2016). They reported the direct α-C (sp^3^)–H acyloxylation of methyl sulfides **43** in the presence of PPh_3_AuCl (0.5 mol%) as a catalyst, bis(acyloxy)iodobenzene **44** as an oxidant, and an acyloxy group source (Figure 6). A variety of thioanisoles **43** functionalized with both electron-rich and -deficient groups reacted with PhI(OAc)_2_ **3** to yield α-thioaryl ester derivatives **45** in moderate to excellent yields. Notably, no product formation was observed in the case of –OH, –NH_2_, and –COOH-substituted thioanisole benzene rings. In the proposed reaction mechanism (Figure 6), the hypervalent iodine reagent **44** acts as a Lewis acid and activates the sulfur atom of the thioether **43** to form sulfonium salt **46** or **47**. Subsequently, sulfonium salt **46** or **47** undergoes elimination in the presence of a gold catalyst to generate intermediate **48** and liberates R^1^COOH. Finally, the nucleophilic attack on the sulfanyl acetate intermediate **48** by–OCOR^1^ gives the desired product **45**.

### Copper

**FIGURE 7:**
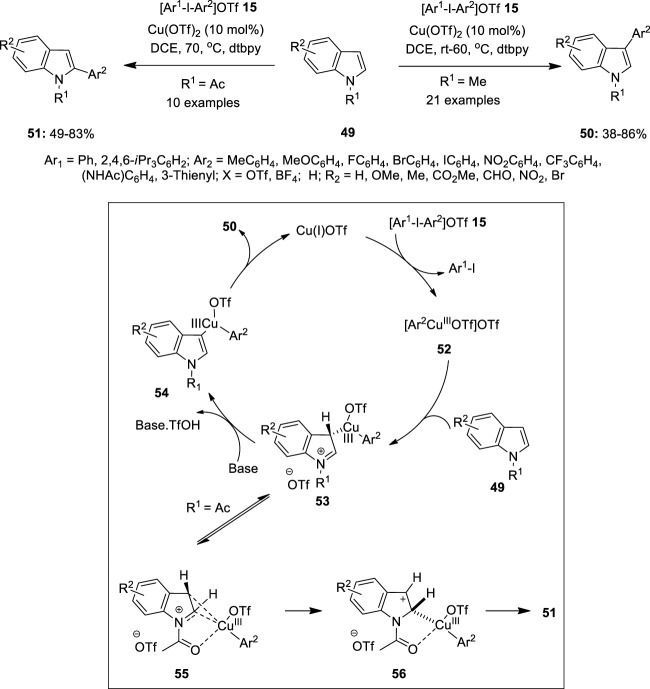
Cu(II)-catalyzed C–H bond arylation of indoles **49** using diaryliodonium salts **15** as a coupling partner.

In recent years, copper has evolved as the most promising alternative to costly metals such as Pd or Rh in metal-catalyzed oxidative coupling reactions. Significant progress has been accomplished in Cu(I)/Cu(III) catalysis and critical experimental studies revealed the clear existence of Cu(III) intermediates in a variety of catalytic transformations. The use of hypervalent iodine reagents as oxidants in copper-catalyzed reactions is a growing area of research. The present section of the review highlights recent examples of copper catalysis mediated by hypervalent iodine reagents. Gaunt et al. demonstrated a site-selective Cu(II)-catalyzed C–H bond arylation of indoles **49** using diaryliodonium salts **15** as a coupling partner (Figure 7) (Phipps et al., 2008). Treatment of (NH)-indoles or *N*-methylated indole **49** with [Ar-I-Ar]OTf salts **15** in the presence of Cu(OTf)_2_ (10 mol%) as a catalyst in dichloroethane yielded 3-arylindoles **50** in 38–86% yields with excellent selectivity. The addition of 2,6-di-tert-butylpyridine (dtbpy) as a base was necessary to avoid indole dimerization. Notably, electron-rich indoles smoothly underwent arylation at room temperature and the corresponding electron-deficient ones required a higher reaction temperature. The reaction scope was also administered with unsymmetrical diaryliodonium salts **15** enabling the efficient transfer of a variety of arenes and heteroarenes aryl motifs at the C3-position of indoles **49**.

The detailed mechanism of this arylation reaction is depicted in Figure 7. The reaction was proposed to initiate with the reduction of the Cu(II) catalyst to Cu(I) by indole **49**, followed by the oxidative addition of diaryliodonium salts **15** to yield an electrophilic Cu(III)-aryl intermediate **52**. The subsequent attack at the C3 position of indole **49** generates intermediate **53**, which aromatizes via C–H bond cleavage forming intermediate **54**. Finally, the reductive elimination of **54** liberates product **50** and re-forms the Cu(I) catalyst. Further reaction of *N*-acetylindole with **49** gave 2-arylindoles **51** selectively in excellent yields. It was speculated that the Cu(III)-aryl intermediate **53** would undergo migration of the Cu(III)-aryl group from the C3 to C2 position of the indole motif and subsequent re-aromatization to afford the C2-arylated indole **51**. Based on the conclusions of Gaunt et al., Wu et al. investigated the site-selective nature of indoles with various directing groups (Wang, et al., 2018). The computational and mass spectrometric studies reveal that a neutral Heck-like mechanism is followed for a weak directing group such as *N*-acetyl indole leading to C_2_ site selection. This is because the electrophilic center tends to coordinate with the most electron-rich C_3_ carbon. However, in the case of indole with a N-P(O)^*t*^Bu_2_ group and N-benzyl-3-pivaloyl indole, a cationic Heck-like reaction is preferred resulting in C_6_ and C_5_ selectivity, respectively (Wang, et al., 2018).

**FIGURE 8:**
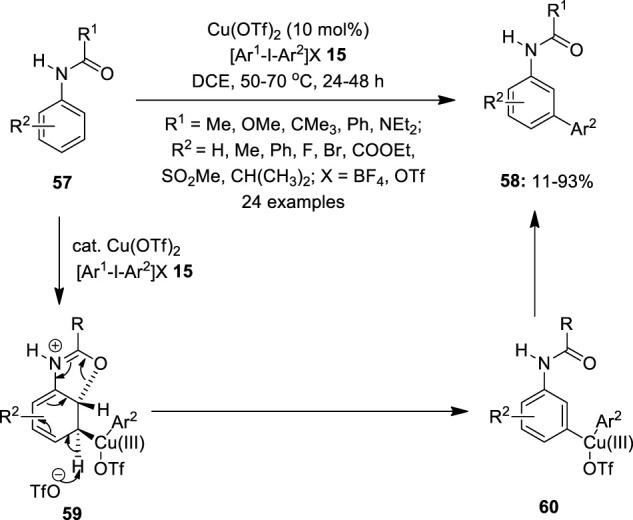
Cu(II)-catalyzed selective *meta* arylation of amides **57** using iodonium salts **15** as an arylating agent.

In continuation, Phipps and Gaunt (2009)designed a simple method to achieve selective *meta* C–H bond arylation by copper catalysis. They treated acetanilide **57** (R^1^ = Me and *R* ^2^ = H) with an arylating agent, Ph_2_IOTf **15** in 1,2-dichloroethane in the presence of 10 mol% Cu(OTf)_2_ as the catalyst and isolated a *meta* arylated product **58** in low yield (Figure 8). With carbamate **57** (R^1^ = OMe and *R* ^2^ = Me) and urea **57** (R^1^ = NEt_2_ and *R* ^2^ = Me), the yield was modest, but with benzamides **57** (R^1^ = Ph and *R* ^2^ = Me) and piv-anilides **57** (R^1^ = CMe_3_ and *R* ^2^ = Me), the yield was excellent. It was observed that no arylation occurred in the absence of an amide group or a copper catalyst. Although the precise mechanism is not given, a possible reaction pathway could involve the activation of an aromatic ring by the highly electrophilic Cu(III)-aryl species, permitting the anti-oxy-cupration of the acetamide carbonyl group across the 2,3 position of the arene ring. This de-aromatization would give a Cu(III)-aryl species at the *meta* position and subsequent re-aromatizing deprotonation, followed by the reductive elimination which would deliver the anticipated *meta* product **58**. While investigating the substrate scope, it was discovered that the electron-rich substrates provided excellent yields for *meta* arylation whereas the electron-deficient ones resulted in albeit lower yields. Remarkably, the reaction also produced good yields from unsymmetrical iodonium salts **15**.

**FIGURE 9:**
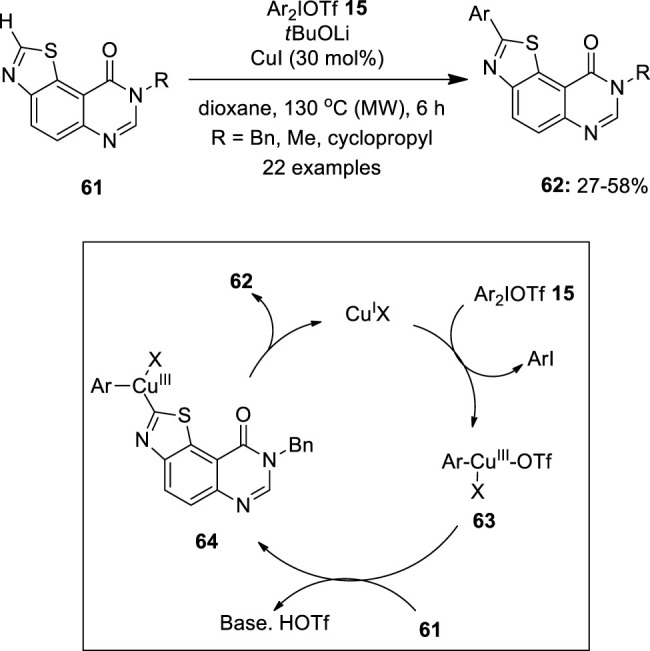
Copper-catalyzed C–H arylation of fused-pyrimidinone derivatives **61** using diaryliodonium triflates **15** as an aryl source.

Recently, C–H arylation of fused pyrimidinone derivatives **61** was developed by Fruit et al using a CuI catalyst loaded with diaryliodonium triflates **15** as an aryl source under microwave irradiation (Pacheco-Benichou et al., 2021) (Figure 9). It was observed that the reactions perform well in CuI as the catalyst in 30 mol% and no product was formed in the absence of a base or catalyst. The addition of 3.5 equivalent of *t*BuOLi as the base and dioxane as the solvent media gave the best C_2_ arylation. The substrate scope showed that aromatic groups with *para* or *meta* CF_3_ groups, and halogens, all offered good yields of arylated compounds **62**. In the case of unsymmetrical diaryliodonium salts **15**, more sterically demanding groupings were shown to migrate preferentially over the others. However, in the absence of the steric effect, the most electron-donating aryl group transfer was favored. Finally, a mechanism was proposed for this reaction, in which a highly electrophilic Cu^III^ intermediate **63** was formed by the oxidative addition of a diaryliodonium salt **15** to Cu^I^ species. Subsequent C–H activation leads to Cu^III^ intermediate **64**, which undergoes reductive elimination to give an arylated product **62** and releases Cu^I^ species for the next catalytic cycle.

**FIGURE 10:**
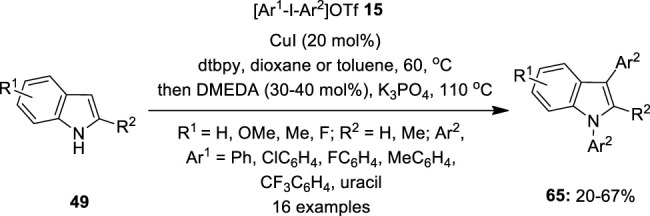
Copper-catalyzed C–H/N−H arylation of indoles **49** with diaryliodonium salts **65** as an aryl source.

Although diaryliodonium salts are gaining much attention as versatile arylating reagents, they always generate 1 equivalent of iodoarene as waste which needs to be separated from the desired product. Within this context, Modha and Greaney (2015) came up with an excellent atom economical protocol for harnessing discrete aryl groups in diaryliodonium salts **15** for double arylation of indoles **49** by copper catalysis

(Figure 10). They performed one-pot copper-catalyzed C−H arylation of indoles **49** followed by *N*-arylation using the *in situ*-generated aryl iodide using the same copper catalyst. The present tandem C−H/N−H process was found to be productive for a range of electron-rich/-poor symmetrical diaryliodonium salts **15**. Further diarylation with unsymmetrical aryl-uracil iodonium triflate **15** was established successfully to furnish a novel indoyl uracil **65** in useful yields.

**FIGURE 11:**
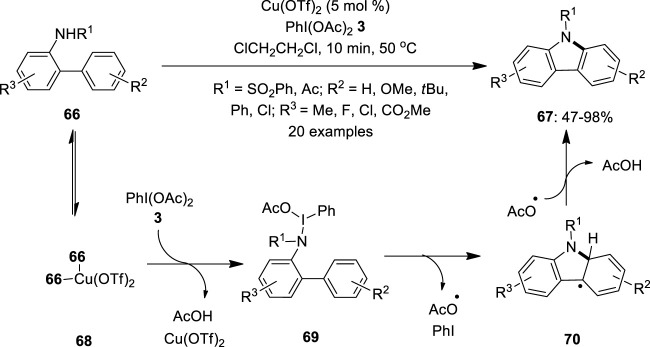
Copper-catalyzed intramolecular cyclization of *N*-substituted amidobiphenyls **66** using PhI(OAc)_2_ **3** as an oxidant.

In 2011, Chang et al. developed a copper-catalyzed synthesis of carbazoles **67** from *N*-substituted amidobiphenyls **66** using hypervalent iodine (III) reagent as an oxidant (Cho et al., 2011). The combination of copper (II) triflate (5 mol%) and PhI(OAc)_2_ **3** significantly promotes this intramolecular oxidative C–N bond formation reaction to produce carbazoles **67** in good to excellent yields (Figure 11). Notably, substrates **66** with electron-rich substituents (*R* ^2^ = OMe, ^*t*^Bu) at the “right” side of the nucleophilic phenyl part provided higher yields whereas those with electron-deficient ones (*R* ^2^ = Cl) resulted in satisfactory product yields. Moreover, a reverse reactivity pattern was observed for the “left” amido-containing aryl part, wherein electron-withdrawing groups facilitate the cyclization. The reaction tolerated both *N*-sulfonyl and *N*-acetylamino substrates; however, lower product **67** yields were obtained in the latter case. The detailed mechanistic investigation revealed that the copper species catalytically activates the iodine (III) oxidant **3** used in the reaction. A plausible reaction pathway is depicted in Figure 11. Initially, 2 equiv. of substrate **66** binds reversibly to the copper to form a tetradentate copper species **68**, followed by the formation of *N*-iodoamido species **69** through the release of acetic acid and Cu(OTf)_2_, which later participates in the next cycles. Furthermore, the *ortho*-phenyl group electrophilically attacks the amido moiety of **69** via a radical path to form a radical species **70**. Finally, the *in situ-*generated acetoxy radical abstracts hydrogen from **70** and delivers carbazoles **67**.

**FIGURE 12:**
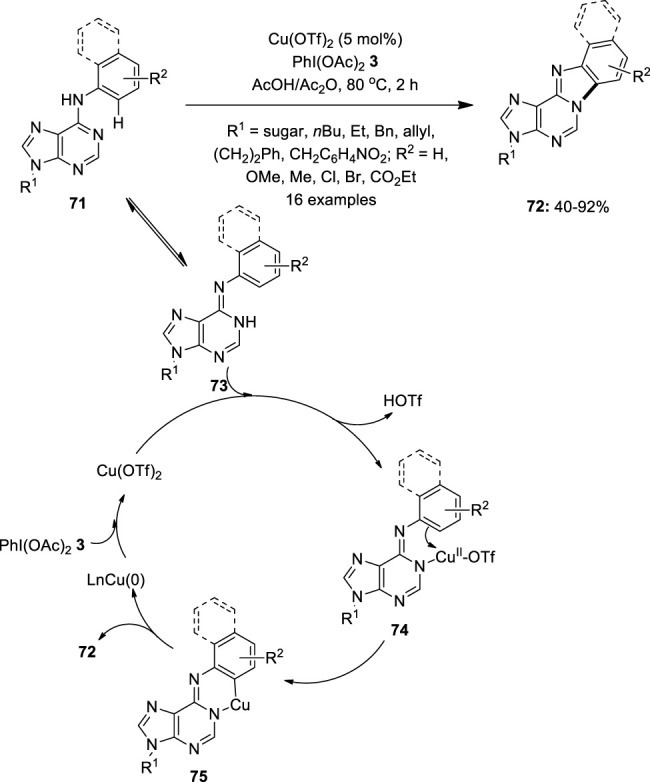
Copper-catalyzed synthesis of multi-heterocyclic compounds **72** from 6-anilinopurine nucleosides **71** using PIDA **3** as an oxidant.

In 2012, Fossey et al. reported the copper-catalyzed C–H activation/intramolecular amination of 6-anilinopurine nucleosides **71** by using PhI(OAc)_2_ **3** as an oxidant (Qu et al., 2012). Among the different catalysts screened, Cu(OTf)_2_ provided the best catalytic activity. This method enabled the facile synthesis of multi-heterocyclic compounds **72** from a variety of purines and its derivatives **71** (Figure 12). Substrates with N9-substituents such as sugar, alkyl, allyl, benzyl, etc. were evaluated successfully for reaction scope. In addition, substrates with electron-withdrawing groups on the aniline ring gave higher yields than those with electron-donating ones. Moreover, steric effect studies showed that *ortho*-substituted substrates **71** were found to be less reactive than those with *para* substituents. A plausible Cu^II^/Cu^0^ catalytic cycle for this intramolecular cyclization is depicted in Figure 12. Initially, Cu(OTf)_2_ coordinates with **73**, followed by an electrophilic substitution process to yield Cu(II) intermediate **75**. Finally, reductive elimination releases the cyclized product **72** and delivers Cu^0^, which can be reoxidized to generate Cu(OTf)_2_.

**FIGURE 13:**
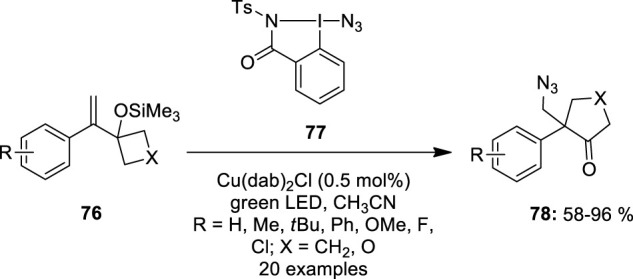
Copper-catalyzed ring expansion of alkene-substituted cyclobutanol derivatives **76** using azidobenziodazolone **77** as an azide precursor.

Waser et al. used azidobenziodazolone **77** as an azide precursor for the ring-expansion of alkene-substituted cyclobutanol derivatives **76** (Alazet et al., 2018). This reaction was performed under photoredox conditions in acetonitrile in the presence of Cu(dap)_2_Cl as a catalyst (Figure 13). The reaction scope was significantly larger, as *para*-substituted styrenes **76** with alkyl-, methoxy-, chloro-, and phenyl groups provided excellent yields. Similarly, *ortho* or *meta-*substituted styrenes **76** produced cyclopentanones **78** in good yields. Boc-protected azetidine and styrene with 1,2 di-substituted olefins, on the other hand, were unresponsive. Unexpectedly, when *para*-CF_3_-substituted styrene **76** was reacted, the only result was epoxide.

**FIGURE 14:**
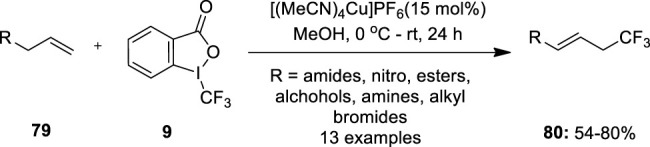
Cu(I)-catalyzed allylic trifluoromethylation of inactivated terminal olefins **79** using Togni’s electrophilic trifluoromethylating reagent **9**.

Parsons and Buchwald (2011) reported Cu(I)-catalyzed allylic trifluoromethylation of terminal alkenes **79** using Togni’s electrophilic trifluoromethylating reagent **9**. This approach has notable advantages such as no harsh reaction conditions, free from toxic or expensive chemicals, and the reagent can be easily recycled. The promising result of these transformations was obtained with 15 mol% of [(MeCN)_4_Cu]PF_6_ as a copper source and methanol as a solvent (Figure 14). Trifluoromethylation of a series of alkenes with variable functional groups such as amides, protected amines, unprotected alcohols, esters, and alkyl bromides was achieved successfully to furnish allyl–CF_3_ products **80** with excellent *E*/*Z* selectivity having an average ratio of 94:6 under these conditions. However, 1,2-disubstituted olefins and branched terminal olefins were found unsuitable substrates because of the formation of undesired regioisomeric product mixtures.

### Platinum

**FIGURE 15:**
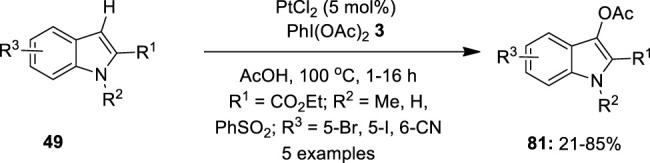
Pt(II)-catalyzed C3-acetoxylation of indoles **49** to afford C3-acetoxylated indoles **81** using PhI(OAc)_2_ **3**.

Platinum has great potential to offer in oxidative coupling reactions owing to its powerful reactivity. Recent developments in this area showed that platinum has remarkable reactivity and excellent selectivity than those with palladium-catalyzed reactions. In this section, we will discuss catalytic reports involving Pt (II)/Pt (IV) intermediates and hypervalent iodine reagents as oxidants. In 2009, Mutule et al. (2009)used a platinum species as an alternative catalyst for the regioselective C–H bond acetoxylation of indoles **49** to prepare biologically important 3-acetoxyindoles **81** via a C–H activation/oxidation strategy. In this reaction, C3-acetoxylation of indoles **49** was performed using 5 mol% PtCl_2_ as the catalyst in the presence of PhI(OAc)_2_ **3** as the terminal oxidant. Optimization studies revealed that other oxidants such as K_2_S_2_O_8_, Cu(OAc)_2_, *t*-BuOOH, and *m*-CPBA were completely inefficient in promoting this acetoxylation reaction. Although the reaction also proceeded well with Pd(OAc)_2_, PtCl_2_ was found more efficient and high yielding. Straightforward access to a variety of 3-acetoxyindole-2-carboxylates **81** was achieved in moderate to good yields. Interestingly, C–H activation/oxidation occurred exclusively at the C-3 position, even in the case of 2,3-unsubstituted indole. The reaction tolerated bromo-, iodo-, and cyano-substituents on the indole moiety under these conditions. Notably, acetoxylation was sensitive to the nature of substituents on the pyrrole ring, particularly, substrates with the *N*-methyl group reacted faster than those with *N*-aryl substituents. In addition, C3-acetoxylation of *N*-unsubstituted indole **49** (*R* ^2^ = H) was also achieved and the anticipated product **81** was isolated in 67% yield (Figure 15).

**FIGURE 16:**
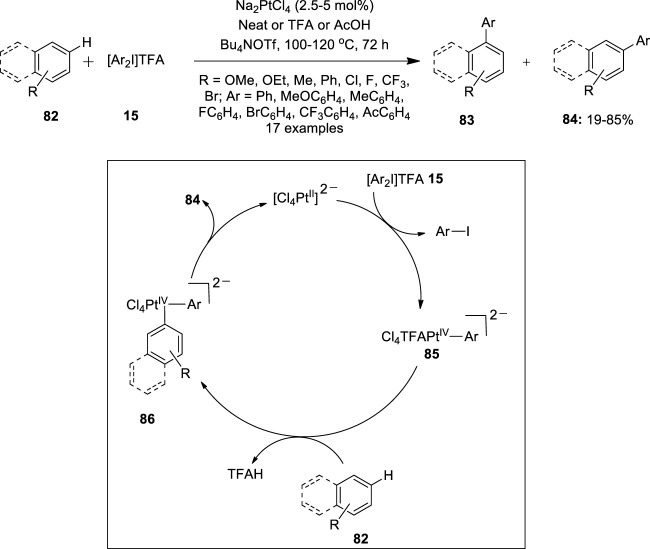
Pt(II)-catalyzed C−H arylation of arenes **82** with diaryliodonium salts **15**.

Furthermore, Sanford et al. used a Na_2_PtCl_4_ catalyst during the C−H arylation of arenes **82** with diaryliodonium salts **15** (Wagner et al., 2013). A variety of aryliodonium salts **15** with *ortho*-, *meta*-, and *para*-substituents were coupled successfully with naphthalene **82** to give biaryl products **84** with high selectivity for isomer **84** over **83** (Figure 16). The reaction produced the best yields in neat naphthalene as revealed by various optimization studies. Notably, C−H arylation of naphthalene **82** showed completely opposite site selectivity on changing the catalyst from Na_2_PdCl_4_ (Hickman and Sanford, 2011) to platinum salt under identical conditions. The site selectivity of this C−H cleavage reaction is predominantly controlled by steric factors, suggesting the feasibility of achieving high selectivity for isomer **84** in Pt-catalyzed naphthalene arylation. Further scope of the arylation reaction was evaluated with electronically diverse arene substrates **82**. Most importantly, the reaction proceeded faster with electron-rich arenes **82** than with electron-deficient ones. A proposed Pt (II)/Pt (IV) catalytic cycle for this reaction is depicted in Figure 16. Initially, [Ar_2_I]TFA **15** oxidizes the Pt (II) catalyst to a Pt (IV)−aryl intermediate **85** followed by subsequent C−H activation of arene **82** to form diaryl Pt (IV) species **86**. Finally, reductive elimination from the Pt (IV) species **86** releases a coupled product **84**. Preliminary mechanistic studies revealed that reductive elimination is the rate-determining step in this Pt-catalyzed reaction.

### Iron

**FIGURE 17:**
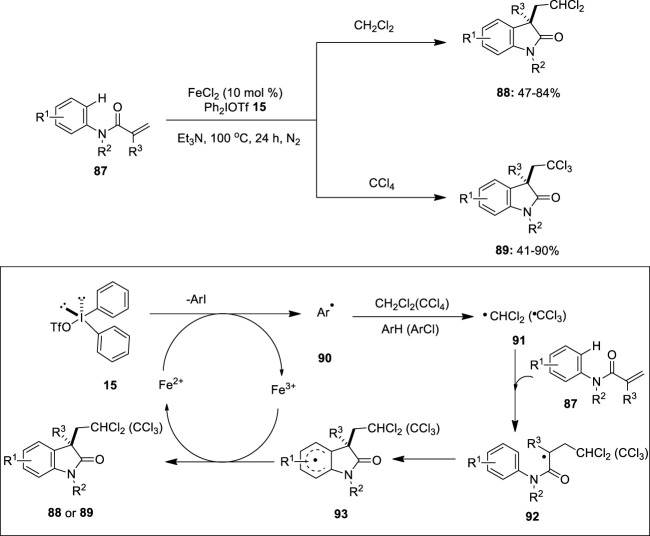
Iron(II)-catalyzed carbodi- and trichloromethylation/cyclization of *N*-arylacrylamides **87** using diaryliodonium salt **15** as an oxidant.

Catalytic cycles involving iron as the catalyst in oxidative coupling reactions are rare. In 2014, Lu and Lu (2014)developed a novel protocol for the iron-catalyzed carbodi- and trichloromethylation/cyclization of *N*-arylacrylamides **87** with dichloro- and tetrachloromethane. This transformation used diaryliodonium salt **15** and triethylamine as an efficient oxidant and a base, respectively. Notably, the reaction of a wide range of *N*-arylacrylamides **87** bearing electron-donating and -withdrawing substituents proceeded smoothly to give the anticipated dichloromethylated oxindoles **88** and trichloromethylated oxindoles **89** in good-to-excellent yields (Figure 17). Substrates **87** with *N*-protection groups such as methyl, ethyl, benzyl, and isopropyl were tolerated; however, no product formation was observed with free *N*-H acrylamide. A plausible reaction mechanism for this reaction is depicted in Figure 17. Initially, diaryliodonium salt **15** generates an aryl radical **90** in the presence of an iron catalyst. Subsequently, aryl radical **90** abstracts hydrogen atoms from CH_2_Cl_2_ or chlorine atoms from CCl_4_ to generate the corresponding dichloromethyl radical (·CHCl_2_) **91** or trichloromethyl radical (·CCl_3_) **91**, followed by the addition to the the carbon–carbon double bond of **87** to form a radical intermediate **92**. Furthermore, the intramolecular cyclization of the radical intermediate **92** gives the intermediate **93**, followed by the single-electron transfer (SET) to the Fe(III) intermediate, and the subsequent base-assisted proton abstraction provides the desired cyclized product **88** or **89**.

### Iridium

**FIGURE 18:**
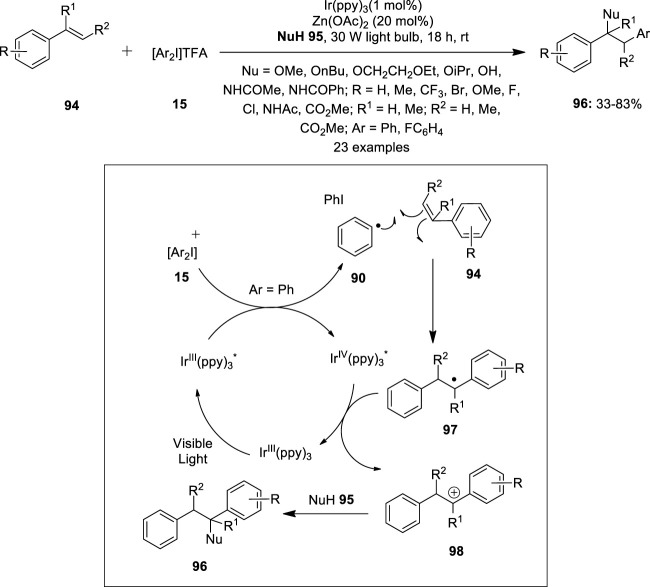
Iridium-catalyzed three-component coupling of styrenes **94** with diaryliodonium salts **15** and heteroatom nucleophiles **95**.

Iridium has received great interest in oxidative cross-coupling reactions involving C–H activation as indicated by various literature reports. In 2013, Greaney et al. described a three-component coupling of styrenes **94** with diaryliodonium salts **15** and heteroatom nucleophiles **95** by photoredox catalysis (Fumagalli et al., 2013) (Figure 18). This method enabled the construction of doubly functionalized phenylethyl scaffolds **96** via simultaneous arylation and C–O or C–N bond formation. Reactions were performed in the presence of Ir(ppy)_3_ (1–5 mol%) as the photoredox catalyst and Zn(OAc)_2_ (20 mol%) as an effective additive. A range of styrene substrates **94** underwent smooth methoxyarylation in degassed methanol affording products **96** in moderate to good yields. The reaction scope was further examined with various heteroatom nucleophiles such as alcohols, water, or nitriles to afford corresponding coupled products **96** in variable yields. The proposed mechanistic pathway is shown in Figure 18. The reaction initiates with the reduction of the diaryliodonium salt **15** with photoexcited Ir(III)* and the resulting aryl radical **90** is trapped by styrene **94** to form a benzylic radical **97**. Subsequently, the oxidation of radical **97** by Ir(IV) generates the cation **98** which can be trapped by an appropriate nucleophile **95** to afford the coupled product **96**.

**FIGURE 19:**
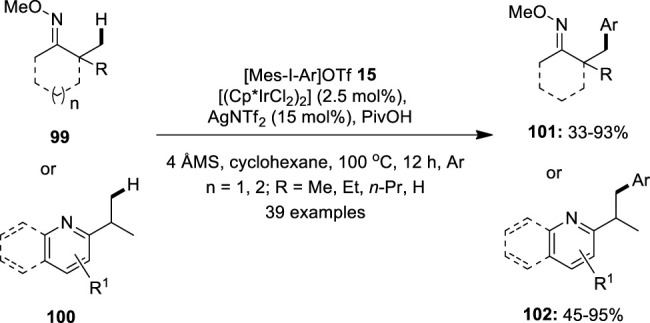
Iridium-catalyzed sp^3^ C–H bond arylation of ketoximes **99** and nitrogen-containing heterocycles **100** with diaryliodonium salts **15**.

Later, Shi et al. demonstrated a direct arylation of the sp^3^ C–H bond in ketoximes **99** and nitrogen-containing heterocycles **100** with appropriate diaryliodonium salts **15** using [(Cp^*^IrCl_2_)_2_] as the pre-catalyst (Gao et al., 2015) (Figure 19). The reaction was performed in the presence of AgNTf_2_ as the halide abstractor and PivOH as the additive. The ketoximes **99** containing α-hydrogens were found to be compatible with this reaction. Furthermore, ketoximes with a six- or seven-membered ring produce the desired product **101** with a high yield. Also, heterocycle-directed sp^3^ C–H bond arylation using pyridine, pyrazine, quinoline, pyrazole, and isoxazole as the directing groups was tested and the respective products **102** were obtained in variable yields. In addition, the reaction scope was successfully extended to the sp^2^ C–H bond arylation of a variety of substituted arenes and olefins under the same optimized conditions.

**FIGURE 20:**
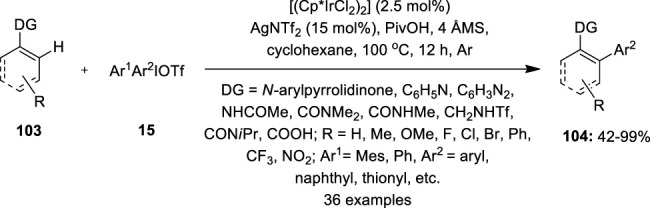
Iridium-catalyzed C–H arylation of arenes and olefins **103** using diaryliodonium salts **15** as an arylating agent.

Moreover, the same catalytic condition was used to arylate the C–H bond of *N*-arylpyrrolidinones **103** and their analogs at the *ortho* position (Gao et al., 2016) (Figure 20). A wide spectrum of *N*-arylpyrrolidinones **103** and diaryliodonium salts **15** having electron-neutral, electron-donating, and halogen-containing motifs were effectively cross-coupled. Substrates containing electron-deficient trifluoromethyl and nitro substituents, on the other hand, produce lesser yields. Interestingly, moderate to excellent yields were obtained when polycyclic and heterocyclic aromatic motifs were coupled. In addition, phenylpyridines, indoles, arenes such as *N*-phenyl amide, benzamides, and sulphonamides, and enamide with both vinylic and allylic C–H linkages and vinyl carboxylic acids were also compatible with the reaction.

**FIGURE 21:**
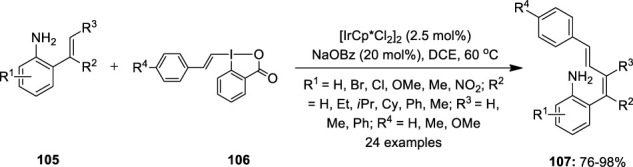
Iridium-catalyzed C–H alkenylation of 2-vinylanilines **105** using alkenyl-3-iodanes **106** as an electrophilic alkene-transfer reagent.

In the next year, NH_2_-directed C–H alkenylation of 2-vinylanilines **105** with alkenyl-3-iodanes **106** as an electrophilic alkene-transfer reagent was developed using the same iridium catalyst (Boelke et al., 2017) (Figure 21). This method enabled the selective synthesis of 1,3-dienes **107** in excellent yields with high *Z* stereoselectivity. Optimization studies revealed that the electron-rich and electron-poor substrates all showed high reactivities similar to that of the un-substituted substrate. Notably, even after altering the different alkyl side chains (*R* ^2^ = Et, *i*Pr, Cy), the corresponding products with high selectivity were retained, whereas the reaction did not occur for highly substituted alkenylanilines **105** (R^3^ = Me, Ph). Further reaction failed with *meta*- and *para-*substituted anilines **105**, thus showing the relevance of the free amino group at the *ortho* position to the exocyclic double-bond as the guiding group. The reaction scope with different vinyl benziodoxolones **106** was also investigated, wherein it was discovered that *p*-toloylvinyl benziodoxolone showed similar reactivity to that of phenyl vinyl benziodoxolone while *p*-methoxyphenylvinyl benziodoxolone displayed lower reactivity.

**FIGURE 22:**
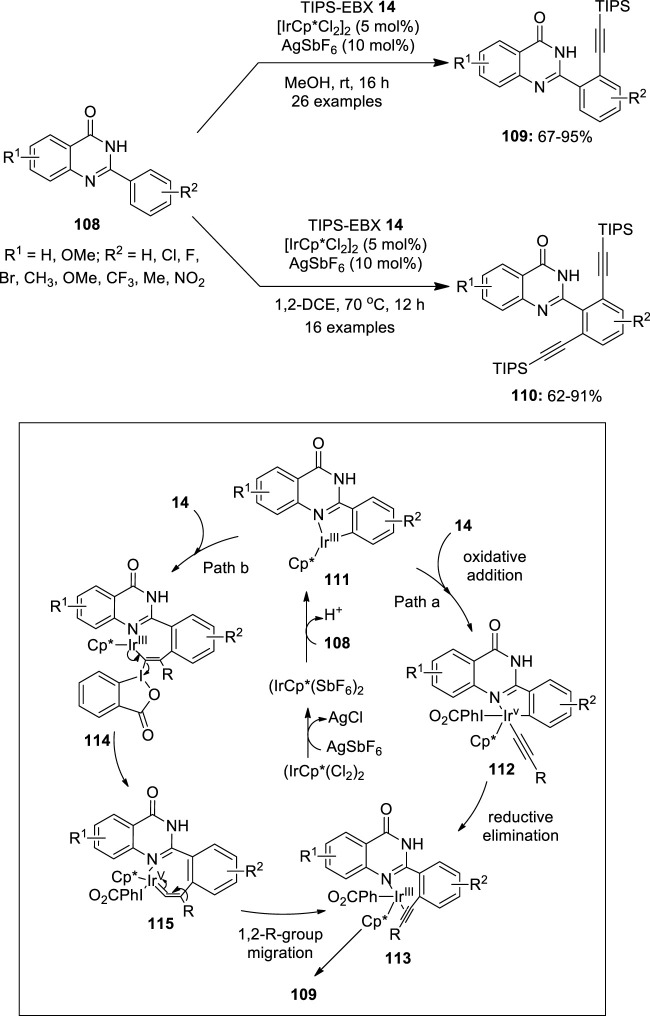
Iridium-catalyzed C–H alkynylation of 2-(hetero)arylquinazolin-4-ones **108** using TIPS-EBX **14** as an alkynylating reagent.

Recently, iridium-catalyzed C–H alkynylation of 2-(hetero)arylquinazolin-4-ones **108** has been investigated using ethynylbenziodoxolone **14** (TIPS-EBX) as an alkynylating reagent (Rohokale et al., 2019) (Figure 22). The reaction was found to be solvent-dependent and an exclusive formation of the monoalkynylated products **109** was observed when a polar protic solvent such as methanol was used as the solvent for this reaction. To form a di-alkynylated product **110** exclusively, dichloroethane at 70°C was used. Notably, selective mono-/dialkynylation of a variety of substrates **108** with different functional groups such as -F, -Cl, -Br, -NO_2_, -OMe, -CF_3_, alkyl, napthyl, etc. proceeded smoothly under these conditions to give mono-/dialkynylated products in good to excellent yields. The tentative mechanism for this reaction is shown in Figure 22. Initially, the dimeric iridium complex reacts with AgSbF_6_ to form a monomeric IrCp*(SbF_6_)_2_ complex, which undergoes a coordinative C−H insertion with 2-arylquinazolin-4-one **108** to give a cyclometalated Ir(III) complex **111**. Authors proposed two pathways for the transfer of the alkyne group from the TIPS-EBX **14** to the aryl ring. In one approach, the oxidative addition of TIPS-EBX **14** results in the formation of an alkynyl-Ir(V) species **112**, which undergoes reductive elimination to yield the crucial Ir(III)-alkyne intermediate **113** (path a). In another path, the complexation of the intermediate **111** with TIPS-EBX **14** followed by the subsequent regioselective migratory insertion of alkyne, resulting in the intermediate **114**, which yields iridium vinylidene species **115** upon the α-elimination of 2-iodobenzoic acid. The intermediate **115** further undergoes concerted 1,2-R-group migration followed by elimination, resulting in the intermediate **113**, a species seen in both pathways a and b. Finally, the dissociation of alkyne from **113** yields the alkynylated product **109** and the active Ir(III) species, which continue the catalytic cycle.

### Nickel

**FIGURE 23:**
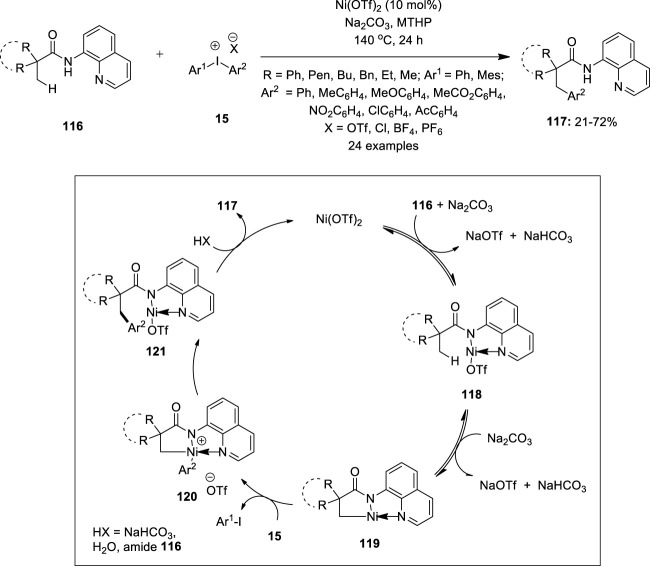
Ni(II)-catalyzed C (sp^3^) –H arylation of aliphatic amides **116** using diaryliodonium salts **15** as an arylating agent.

Iyanaga et al. described the first example of Ni(II)-catalyzed 8-aminoquinoline directed C (sp^3^) –H arylation of aliphatic amides **116** using diaryliodonium salts **15** as an arylating agent (Iyanaga, et al., 2014) (Figure 23). The reaction used Ni(OTf)_2_ (10 mol%) as the catalyst and 4-methyltetrahydro-2*H*-pyran (MTHP) as the solvent system. Moreover, Na_2_CO_3_ as the base resulted in high product yields. Under this condition, β-arylation products **117** were obtained in good to excellent yields with broad functional group compatibility. Diaryliodonium triflates **15** with electron-donating aryl groups produced higher product yields than those with electron-withdrawing groups. Amides with no α-hydrogen reacted exclusively at the methyl group whereas those with methylene or benzene C–H bonds were not arylated. The proposed mechanistic pathway for the reaction is shown in Figure 23. Initially, amide **116** co-ordinates with the Ni center and followed by ligand exchange forms the Ni complex **118**, which further undergoes reversible cyclometalation to give the intermediate **119** *via* a CMD mechanism. The oxidative addition of the diaryliodonium salt **15** to the intermediate **119** generates a high-valent Ni(IV) complex **120** and aryl iodide. Finally, reductive elimination of the complex **120** and subsequent protonation affords the desired arylation product **117** along with the regeneration of Ni(II) for the next catalytic cycle.

### Ruthenium

**FIGURE 24:**
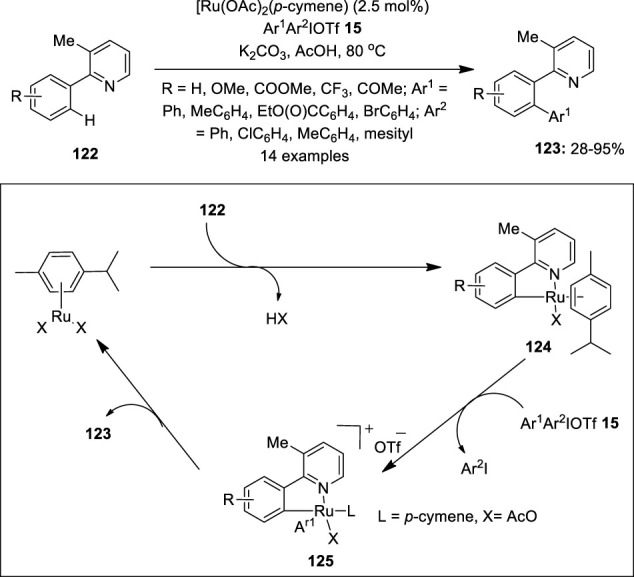
Ru^2+^-catalyzed arylation of 2-arylpyridines **122** with Ar^1^Ar^2^IOTf **15** as an arylation reagent.

In 2014, Chatani et al. reported the Ru(II)-catalyzed C–H arylation of 2-arylpyridine derivatives **122** using diaryliodonium salts **15** as an arylating reagent (Ho et al., 2014). The reaction used [Ru (OAc)_2_ (*p*-cymene)] as an active catalyst, K_2_CO_3_ as a base, and AcOH as a solvent (Figure 24). The substrates’ scope revealed that electron-rich 2-arylpyridines **122** gave the corresponding product **123** in lower yields than those with electron-deficient ones. A variety of mixed diaryliodonium salts **122** were used, particularly electron-rich and sterically less demanding aryl groups are selectively transferred to give **123** in useful yields. Notably, the reaction yielded single arylated products and tolerated a broad range of functional groups. The proposed reaction mechanism is discussed in Figure 24. Initially, 2-phenylpyridine **122** reacts with a Ru(II) catalyst to form a cyclometalated ruthenium complex **124**, which upon oxidative addition with Ar^1^Ar^2^IOTf **15** gives the Ru(IV) complex **125**. Finally, the complex **125** undergoes reductive elimination to give an arylated product **123** along with the regeneration of the Ru(II) catalyst. Another example of Ru-catalyzed C–H arylation of arenes and hetero-arenes using diaryliodonium salts **15** was reported previously by Xiao et al. (Liu et al., 2013).

## Conclusion

A transition metal-catalyzed direct oxidative coupling reaction has been identified as a straightforward route for C–C and C–heteroatom bond formation. This strategy is most elegant, economic, and environmentally friendly than traditional cross-coupling reactions. For many years, most of these coupling reactions used palladium as a catalyst, but recent studies showed other transition metal catalysts such as gold, copper, platinum, iron, nickel, and ruthenium as potential alternative candidates for these reactions. Hypervalent iodine reagents have evolved as versatile oxidants and functional group transfer reagents in this area particularly because of their excellent oxidizing and electrophilic properties. This has led to the rapid development in transition metal-catalyzed homo/cross-coupling, arylation, alkenylation, alkynylation, acyloxylation, and alkene functionalization reactions. PhI(OAc)_2_ is the most commonly used oxidant whereas diaryliodonium salts and TIPS-EBX are widely used as arylating and alkynylating reagents, respectively, in these reactions. Despite the great success of hypervalent iodine reagents, the main drawback associated with them is the stoichiometric generation of byproducts, which needs to be addressed in future. In addition, there exists limited scope of hypervalent iodine (III) reagents for functional group transfer reactions. Moreover, the development of newer hypervalent iodine compounds that can be readily recycled or regenerated would be an active area of research to expand the versatility of this reaction manifold.
